# Identification of a Novel Heterozygous Mutation in the *EIF2B4* Gene Associated With Vanishing White Matter Disease

**DOI:** 10.3389/fbioe.2022.901452

**Published:** 2022-07-04

**Authors:** Yun Tian, Qiong Liu, Yafang Zhou, Xiao-Yu Chen, Yongcheng Pan, Hongwei Xu, Zhuanyi Yang

**Affiliations:** ^1^ Department of Geriatrics, Xiangya Hospital, Central South University, Changsha, China; ^2^ National Clinical Research Center for Geriatric Disorders, Xiangya Hospital, Central South University, Changsha, China; ^3^ Department of Neurology, Xiangya Hospital, Central South University, Changsha, China; ^4^ Key Laboratory of Hunan Province in Neurodegenerative Disorders, Central South University, Changsha, China; ^5^ Department of Neurosurgery, Xiangya Hospital, Central South University, Changsha, China

**Keywords:** vanishing white matter disease, EIF2B4, heterozygous missense mutation, loss of function, targeted gene capture sequencing panel

## Abstract

Vanishing white matter disease (VWM) is one of the most common childhood inherited leukoencephalopathies with autosomal recessive inheritance. Mutations in five genes, *EIF2B1-5*, have been identified as the major cause of VWM. In this study, a targeted gene capture sequencing panel comprising 160 known pathogenic genes associated with leukoencephalopathies was performed in a large Han Chinese family affected by adult-onset VWM, and a novel heterozygous missense mutation (c.1337G > A [p. R446H]) in *EIF2B4* (NM_001034116.2) was detected. Further functional studies in HEK 293 cells showed dramatically reduced EIF2Bδ protein levels in the mutated group compared with the wild-type group. This study revealed that a heterozygous missense mutation (c.1337G > A [p. R446H]) in *EIF2B4* was potentially associated with the adult-onset mild phenotype of VWM. In contrast to previous reports, autosomal dominant inheritance was also observed in adult-onset VWM.

## Introduction

Vanishing white matter disease (VWM), also known as childhood ataxia with central nervous system hypomyelination (CACH), is one of the most common childhood inherited leukoencephalopathies. It is an autosomal recessive disease characterized by progressive ataxia, spasticity, and leukoencephalopathy. The age of the VWM onset varies from before birth to adulthood, with most patients presenting with an onset between the ages of 2 and 6 years (Wu et al., 2009). Mental function is better preserved than motor function. For instance, intellectual problems are relatively rare in VWM, and psychiatric symptoms may occur as an initial manifestation in adult-onset patients. Episodic neurological regression and coma provoked by fever and head trauma usually appear during the progressive course of the disease (Van Der Knaap et al., 1997; Bugiani et al., 2010). Some female patients also experience ovarian failure (Fogli et al., 2003). The clinical phenotypes of VWM vary between studies; notably, the earlier the age of onset, the more severe the disease (Hamilton et al., 2018). Most individuals with VWM display a severe progressive phenotype in early childhood that may end in early death (Van Der Knaap et al., 2003), while a few instances have developed in adulthood and presented much milder progression (Pronk et al., 2006). Compared to early childhood-onset VWM, adult-onset VWM has rarely been reported. The clinical course of adult-onset VWM may be milder and slower, but it is still progressive, and many patients ultimately lose the ability to perform daily living activities (Labauge et al., 2009). Typical brain magnetic resonance imaging (MRI) shows extensive leukoencephalopathy with diffuse fluid-like signals as a strong indicator of VWM (Van Der Knaap et al., 1999).

The eIF2B complex, which consists of five subunits (eIF2Bα, β, γ, δ, and ε) encoded by *EIF2B1-5* genes, is critical for the initiation of protein translation (Pavitt, 2005; Lorsch and Dever, 2010; Hinnebusch, 2011). Defects in any of these five subunits impair protein synthesis by reducing eIF2B activity (Pavitt et al., 1997; Kimball et al., 1998; Sonenberg and Hinnebusch, 2009; Elsby et al., 2011). Mutations in the *EIF2B1-5* genes have been identified as the major cause of VWM and follow an autosomal recessive inheritance mode (Leegwater et al., 2001; Van Der Knaap et al., 2002). Patients diagnosed with VWM have been reported to have either homozygous or compound heterozygous variants in one of the aforementioned five genes, while very few harbor heterozygous mutations (Wu et al., 2009; Zhang et al., 2015). To date, more than 150 mutations in EIF2B have been reported (Zhang et al., 2015). Most of the reported patients with VWM are Caucasian, while Han Chinese patients with *EIF2B* gene mutations have rarely been reported. Moreover, none of the Chinese affected individuals had adult-onset (Wu et al., 2009; Zhang et al., 2015).

In this study, we performed targeted gene capture sequencing followed by Sanger sequencing validation in a Chinese family affected by adult-onset VWM and identified a novel heterozygous missense mutation (c.1337G > A [p. R446H]) in *EIF2B4*, which led to dramatically reduced EIF2B4 protein levels when overexpressed in HEK 293T cells.

## Materials and Methods

### Subjects

A Han Chinese VWM-affected family and healthy controls were recruited for this study. All study participants were carefully evaluated by at least two trained neurologists. VWM was diagnosed according to the clinical manifestation of motor and/or cognitive dysfunction accompanied by typical leukoencephalopathy with diffuse fluid-like signals on brain MRI. Clinical data and blood samples were collected from all participants.

This study was approved by the Ethics Committee of Xiangya Hospital of Central South University in China. Written informed consent was obtained from all the participants.

### Mutation Screening

A QIAGEN kit was used to extract genomic DNA from peripheral blood leukocytes. To screen for mutations in the probands, we used a targeted gene capture sequencing panel composed of 160 genes reported to be associated with leukoencephalopathies, including *AARS2*, *ABAT*, *ABCD1*, *ACOX1*, *ACTA2*, *ADH1C*, *AIMP1*, *ALDH3A2*, *APBB2*, *ARSA*, *ASPA*, *BCKDHA*, *BCKDHB*, *BCS1L*, *CHM*, *CLCN2*, *COA5*, *COX15*, *COX6B1*, *CSF1R*, *CTC1*, *CYP27A1*, *DARS2*, *DBT*, *DDC*, *DLD*, *DNAJC13*, *DNAJC6*, *ECM1*, *EIF2B1*, *EIF2B2*, *EIF2B3*, *EIF2B4*, and *EIF2B5* (Supplementary Table S1). Biotinylated RNA probes were used to capture the known DNA sequences from the GRCh37/hg19 human reference sequence. After QC was used for quality control of sequencing data, low-quality reads were filtered out. The sequence reads were then aligned to the GRCh37/hg19 human reference genome using the Burrows–Wheeler Aligner (BWA, v.0.7.15). Raw variant calling was performed using the Genome Analysis Toolkit (GATK, v.3.2). The variants, including single nucleotide variants (SNVs) and indels (insertions and deletions), were annotated using ANNOVAR. Public databases, including 1,000 human genome datasets (1,000 Genomes) and the Genome Aggregation Database dataset (gnomAD, v2.11), were used to screen mutations among previously obtained SNVs and indels. The variants were filtered in accordance with allele frequencies, with minor allele frequencies (MAFs) < 0.001. Damaging missense and splicing mutations were predicted using the database for nonsynonymous SNPs’ functional prediction descriptions (dpNSFP, v.4.0), Sorting Intolerant from Tolerant (SIFT) (Alanazi et al., 2011) and Polymorphism Phenotyping v2 (PolyPhen-2) (Adzhubei et al., 2010). Amino acid conservation around mutated positions was evaluated using T-Coffee (v.11.00) (Di Tommaso et al., 2011), to blast multiple sequences of eIF2Bδ proteins from different species. A 3D structural protein model was predicted using Phyre2 (Kelley et al., 2015). In addition, reported mutations were screened using the Human Gene Mutation Database (HGMD) and the ClinVar database. All variants were classified based on the sequence variant standards and guidelines of the American College of Medical Genetics and Genomics (ACMG) (Li et al., 2017). Further Sanger sequencing validation of family members was also performed.

### Expression Plasmids

The wild-type *EIF2B4* plasmid was generated by cloning the complementary DNA (cDNA) of *EIF2B4* (NM_001034116.2) into mammalian expression vectors (pcDNA3.1) with a FLAG tag at the C-terminus. *EIF2B4* variants (p. R374C and p. R446H) were generated using a QuickChange II site-directed mutagenesis kit (Stratagene, 200523). The authenticity of all constructs was confirmed using Sanger sequencing.

### Antibodies

The primary antibodies used in this study included FLAG (Sigma, F1804), vinculin (Cell Signaling Technology, 13901), and β-actin (Cell Signaling Technology, 4967L). The secondary antibodies were donkey anti-rabbit and donkey anti-mouse antibodies (Jackson ImmunoResearch).

### Cell Culture and Transfection

Human embryonic kidney (HEK) 293 cells were grown at 37°C under 5% CO2 in Dulbecco’s modified Eagle’s medium supplemented with 10% fetal bovine serum and 100 U/mL of penicillin/streptomycin. Expression plasmids were transfected into cells using Lipofectamine 2000 reagent (Invitrogen), according to the manufacturer’s protocol. The cells were collected for Western blotting 48 hours after transfection.

### Western Blot

Transfected cells were lysed in ice-cold RIPA buffer containing a protease inhibitor cocktail and PMSF. The protein samples were separated using 10% SDS-PAGE and transferred onto a PVDF membrane (Millipore). After blocking, the blots were probed overnight with the appropriate antibodies. Blots were developed using an ECL prime chemiluminescence kit (GE Healthcare). Images were obtained using ChemiDoc XRS+ with Image Lab software (Bio-Rad).

## Results

### Clinical Assessment of the VWM-Affected Family

**FIGURE 1:**
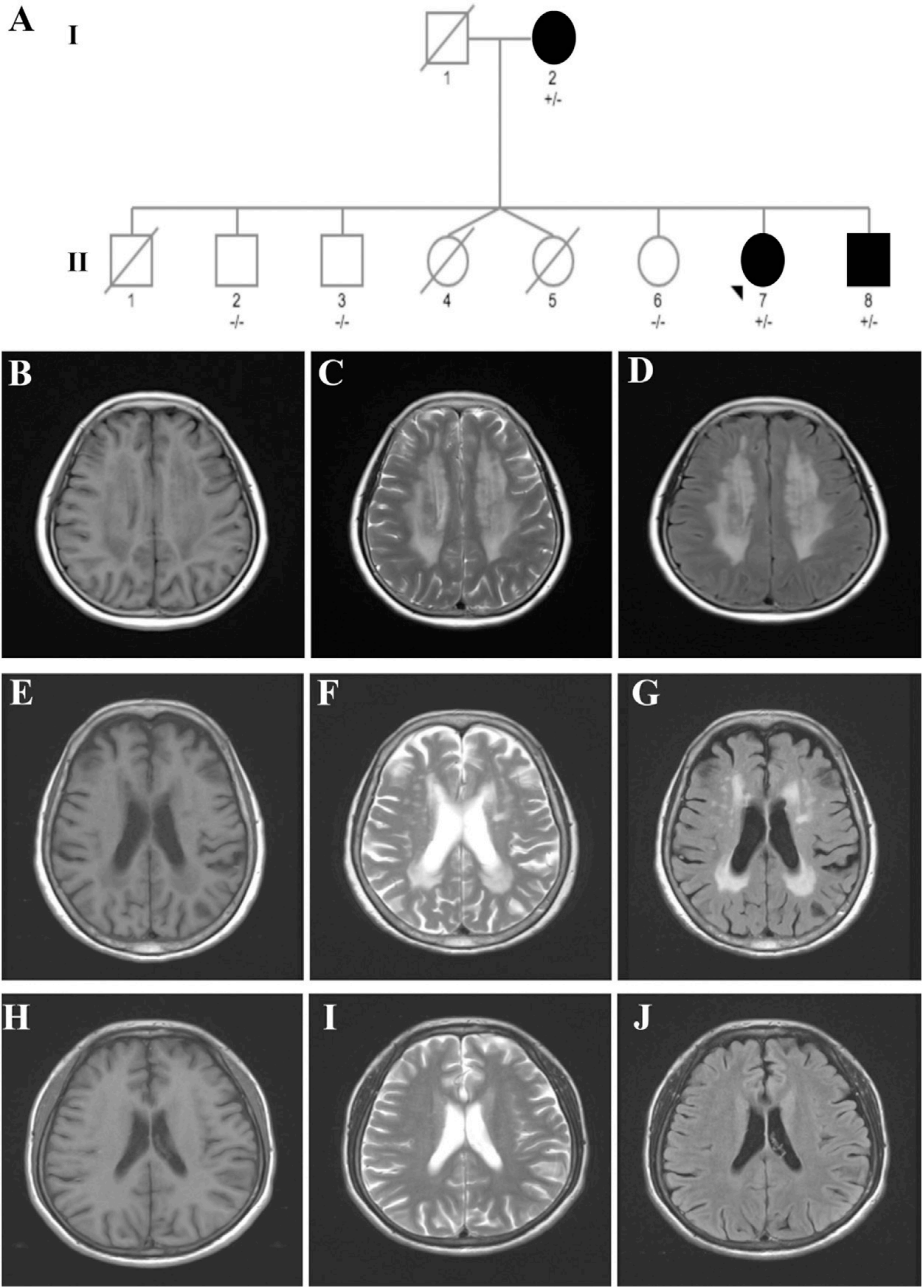
Pedigree of a family affected by VWM. **(A)** Pedigree of the VWM-affected family. **(B–D)** Brain MRI of the proband (case II-7) indicating typical symmetric and diffuse periventricular leukoencephalopathy and a cerebrospinal fluid-like signal within the area on T1-weighted **(B)**, T2-weighted **(C)**, and flair images **(D)**. **(E–G)** Brain MRI of the mother (case I-2) showing moderate leukoencephalopathy. **(H–J)** Brain MRI of the brother (case II-8) revealed no leukoencephalopathy.

The pedigree with VWM included three affected individuals (Figure 1A).

The proband case (case II-7) was a 53-year-old woman who presented with progressive cognitive decline for 3 years. At the age of 50, she experienced memory loss and became easily lost in new places. Four days before admission, her symptoms worsened after staying up late. Patients with ovarian failure or other medical history were excluded. The neurological examination revealed memory loss, a decline in calculation ability, language dysfunction, and visuospatial disorders. Her Mini-Mental State Examination (MMSE) score was 14, and the Montreal Cognitive Assessment (MoCA) Scale score was 12. Magnetic resonance imaging (MRI) revealed symmetric and diffuse leukoencephalopathy in the periventricular and cerebrospinal fluid-like signal within the area on T1-weighted, T2-weighted, and flair images (Figures 1B–D).

The mother of the proband (case I-2), an 85-year-old woman, was referred to our hospital with a 20-year history of cognitive dysfunction. Neurological examination revealed mild cognitive impairment. Her MMSE score was 18, with 0-year education. Moderate leukoencephalopathy was observed on her brain MRI, while no fluid-like signal was observed (Figures 1E–G).

The younger brother of the proband (case II-8) was a 50-year-old man who presented with abnormal behavior at the age of 44. He had been diagnosed with schizophrenia 5 years before and had received risperidone for treatment. He had been experiencing memory loss since childhood and was unable to walk until 7 years of age. However, no leukoencephalopathy was observed on his brain MRI (Figures 1H–J).

It is noteworthy that case II:1 died at the age of 3 years for unknown reasons. Coincidently, cases II-4 and 5, a pair of twin sisters, died a few days after birth. It is possible that these three patients may have been affected by VWM and died young. However, further confirmation was difficult because we were unable to obtain their exact medical history and DNA samples.

### Identification of Novel Heterozygous Mutations in *EIF2B4*

**FIGURE 2:**
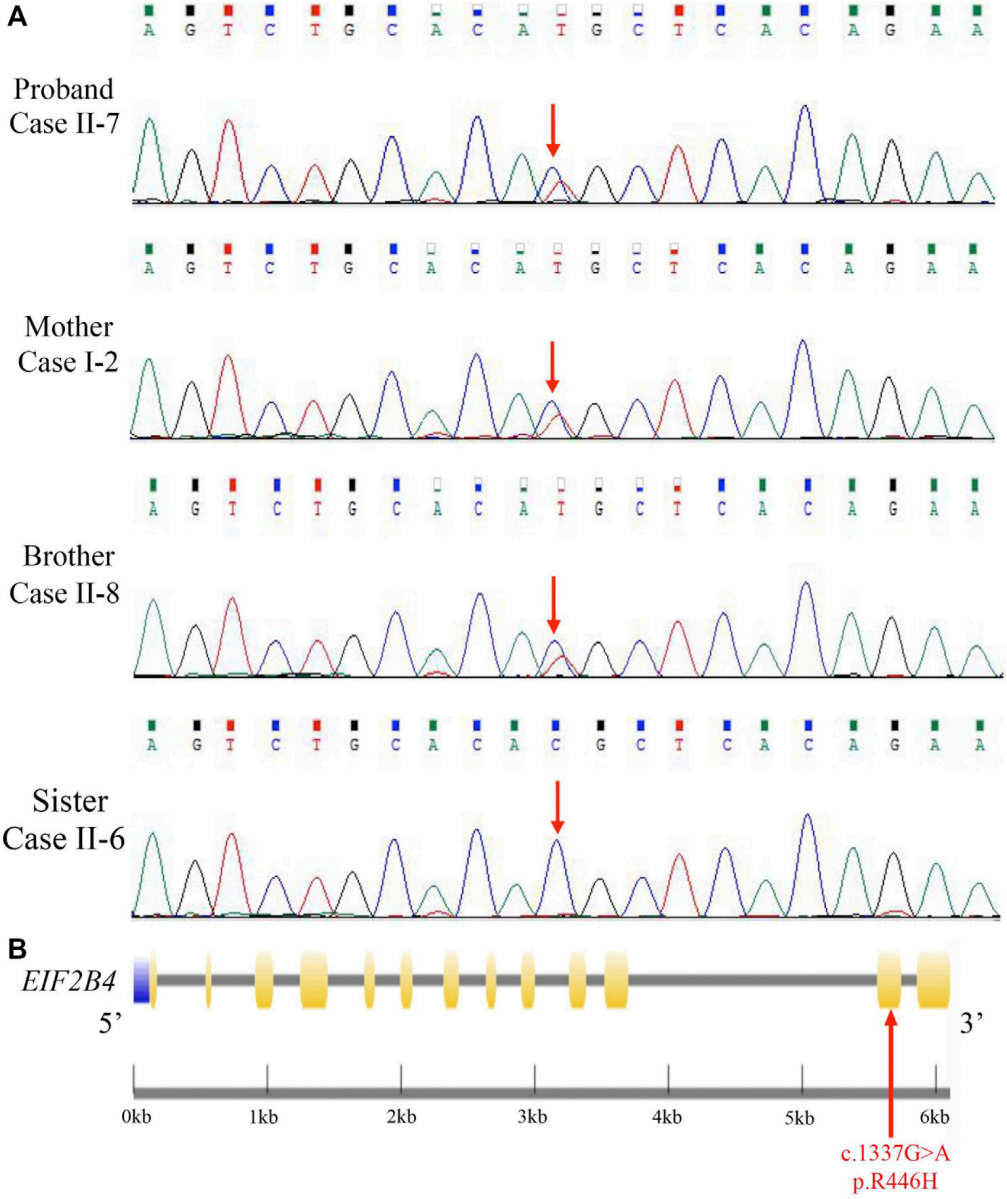
Identification of a novel heterozygous *EIF2B4* mutation. **(A)** Sanger sequencing validated a missense mutation (c.1337G > A [p. R446H]) in the *EIF2B4* gene in family members. **(B)** Schematic presentation of the *EIF2B4* gene with an R446H substitution located in exon 12. Purple: upstream; yellow: exons; black line: introns.

Mutation screening was performed in the proband by using a targeted gene capture sequencing panel composed of 160 genes associated with leukoencephalopathies. A novel heterozygous missense mutation (c.1337G > A [p. R446H]) in *EIF2B4* (NM_001034116.2) was identified, but no other potential variants in reported genes associated with leukoencephalopathies were detected. Sanger sequencing validation showed that none of the cases had other pathogenic or likely pathogenic mutations in this gene (Figure 2A).

The *EIF2B4* gene is located on chromosome 2p23.2, encoding a 67-kD protein. This mutation lies in exon 11 (Figure 2B), resulting in an amino acid substitution of arginine to histidine (R446H) at the C-terminus of the eIF2Bδ protein. The majority of mutations reported to contribute to VWM or ovarioleukodystrophy (OLD) are missense mutations in the *EIF2B4* gene (Fogli et al., 2004; Scali et al., 2006), but this mutation has not been reported in the HGMD and ClinVar databases. We reanalyzed the sequencing data from 12 previously unrelated patients with non-vascular leukoencephalopathies, and no other patients were found to carry the R446H mutation. We further checked whole-exome sequencing data from 1,017 patients with neurological disorders, and none of them carried this mutation. The p. R446H substitution is absent from the human population database 1,000 Genomes, and the frequency of the p.R446H variant is 0.00006 in the gnomAD.

**FIGURE 3:**
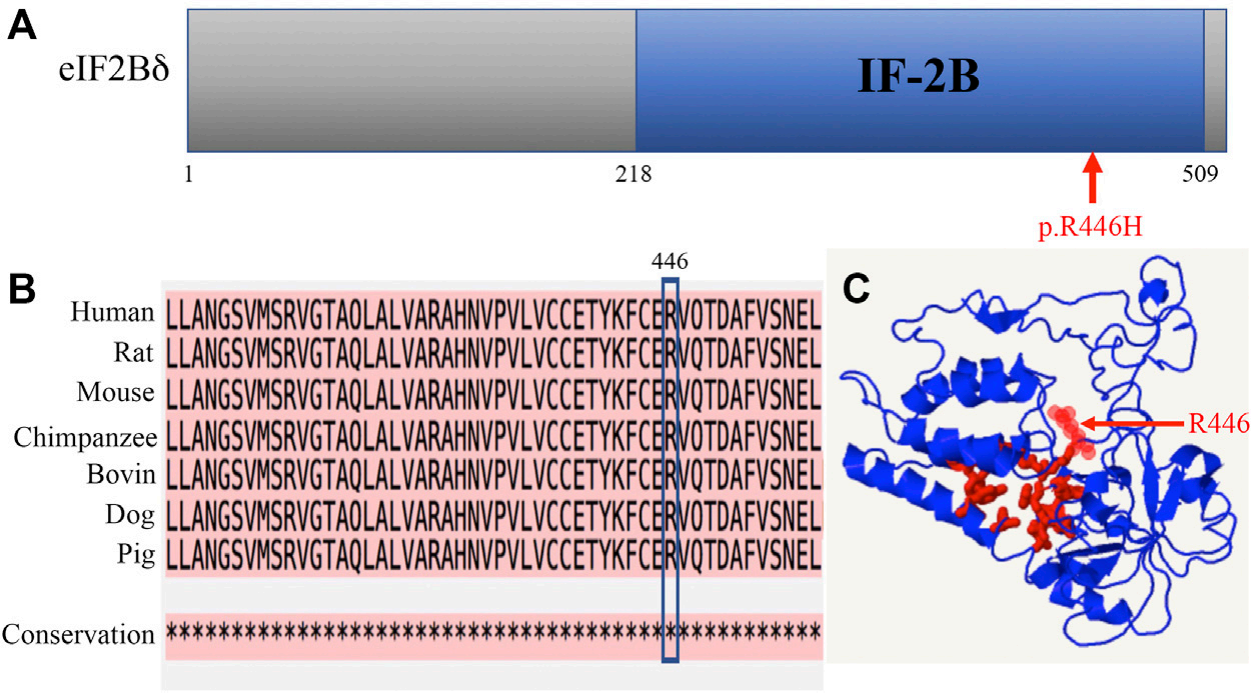
Amino acid R446 is highly conserved among mammals. **(A)** Location of the R446H mutation in the eIF2Bδ protein. **(B)** Amino acid sequence alignments across species indicate high conservation around residue R446. * fully conserved. **(C)** 3D structural prediction of the eIF2Bδ protein. Red: pocket; red arrow: residue R446.

The p.R446H substitution was predicted to affect protein function by SIFT and was expected to be probably damaged by PolyPhen-2. The mutation lies in the IF-2B domain of the eIF2Bδ protein (Figure 3A). IF-2B resembles a domain that belongs to the initiation factor 2 subunit family, which includes IF-2Bα, β, and δ subunits from eukaryotes and some other proteins of unknown function from archaebacteria and prokaryotes (Kyrpides and Woese, 1998). The amino acid sequence alignments remained highly conserved across vertebrates within this region (Figure 3B), indicating that these residues play a critical role in eI2Bδ function. The 3D structural protein model revealed that residue R446 is located close to the predicted pocket region (Figure 3C), which might be functionally important.

Furthermore, the *EIF2B4* (c.1337G > A [p. R446H]) mutation was found in all affected individuals but not in unaffected family members of the pedigree (Figure 2A and Supplementary Figure S1). According to the ACMG guidelines, the novel R446H substitution is classified as a variant of uncertain significance (VUS), indicating that a heterozygous (c.1337G > A [p. R446H]) mutation in the *EIF2B4* gene may be associated with VWM.

### The p. R446H Substitution Significantly Reduces eIF2Bδ Protein Levels

**FIGURE 4:**
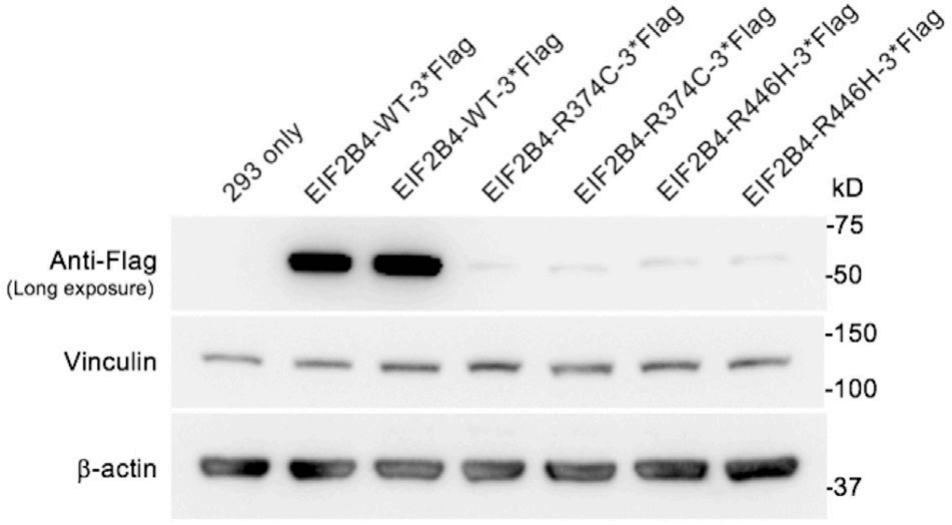
Expression of wild-type and mutant eIF2Bδ in transfected HEK 293 cells. The levels of eIF2Bδ−R374C and R446H were significantly reduced compared with the wild-type protein.

To evaluate the pathogenicity of the R446H substitution, vectors containing wild-type and mutant *EIF2B4* (R374C and R446H, respectively) with a C-terminal flag tag were transfected into HEK 293 cells for functional *in vitro* studies. The R374C variant is also a missense mutation that has been previously reported to be related to VWM before (Van Der Knaap et al., 2002). The expression levels of R374C and R446H were measured using an anti-flag antibody and were found to significantly decrease compared to wild type (Figure 4). These results suggest that both p.R446H and p.R374C substitutions lead to haploinsufficiency of eIF2B, δ, which may contribute to VWM.

## Discussion

To date, at least 250 patients with VWM and 150 mutations in *EIF2B* genes have been reported (Zhang et al., 2015). Most *EIF2B* gene mutations have been reported in Caucasian populations, and the only study that was conducted in a Chinese population reported 34 patients with VWM and 37 *EIF2B* gene mutations (Zhang et al., 2015). Among all reported *EIF2B* mutations, *EIF2B4* gene mutations were observed in both Caucasian and Chinese patients with frequencies of 17% and 22%, respectively (Scali et al., 2006; Zhang et al., 2015). Rare mutations in the *EIF2B4* gene have been reported to account for 10–15% of Caucasian patients with VWM (Fogli et al., 2004; Pronk et al., 2006) and 18% of Han Chinese patients with VWM (Zhang et al., 2015). Most of the reported pathogenic mutations are missense mutations, which are mainly distributed in the highly conserved C-terminal region of eIF2B (Pavitt, 2005).

Patients with VWM with *EIF2B4* gene mutations are either homozygous or compound heterozygous, and the majority develop symptoms before adulthood. Consistent with patients with VWM with other *EIF2B* gene mutations, *EIF2B4* gene mutations are usually associated with motor dysfunction, rather than cognitive decline, as cognitive function is preserved relatively well. Some patients present motor development delays, speech delays, or school difficulties before the disease onset (Fogli et al., 2004; Ohlenbusch et al., 2005; Zhang et al., 2015). Only two adult-onset patients with *EIF2B4* gene mutations have been previously reported, both of which were compound heterozygous variants. Furthermore, both patients experienced cognitive impairment and motor symptoms, with mild progression (Kanbayashi et al., 2015).

In our study, we identified a novel heterozygous missense mutation (c.1337G > A [p. R446H]) in the *EIF2B4* gene in a family with a milder form of adult-onset VWM with cognitive decline or abnormal behavior as their chief complaint. The younger brother of the proband (case II-8) had motor developmental delays in early childhood. Unlike previously reported adult-onset cases of *EIF2B4* mutations, no motor symptoms were observed in this study.

The R446H variant identified here is located in a highly conserved region within the C-terminus of eIF2Bδ. The eIF2Bδ protein is ubiquitously expressed in human organs and is one of the five subunits of the eIF2B complex. The eIF2B complex is involved in translation initiation, where its guanine nucleotide exchange (GEF activity) turns inactive GDP-eIF2 to an active GTP-bound form (Leng et al., 2011). Furthermore, the eIF2B complex is composed of two α, β, and δ subunits in the central region, with one γε heterodimer on each side (Kashiwagi et al., 2019). The catalytic center of the γε subcomplex interacts with eIF2 and exhibits GEF activity (Pavitt, 2005), while the α_2_β_2_δ_2_ subcomplex recognizes phosphorylated eIF2α and inhibits GEF activity (Kashiwagi et al., 2019). The C-terminus of the δ subunit shares a similar sequence with the α and β subunits, annotated as the initiation factor 2 subunit family, which suggests that these three subunits may interact with each other to regulate the eIF2B complex (Kyrpides and Woese, 1998; Pavitt, 2005).

Our functional study revealed that overexpression of the R446H variant resulted in a significant reduction in eIF2Bδ levels, which may reduce the activity of the eIF2B complex and impair its physiological function (Bugiani et al., 2010). It is reasonable to postulate that the R446H variant may cause VWM, even in a heterozygous status, by reducing the activity of eIF2B in patients.

In conclusion, our work indicated that a heterozygous missense mutation (c.1337G > A [p. R446H]) in the *EIF2B4* gene might be associated with an adult-onset milder form of VWM, and the genetic and clinical spectrum of VWM was extended. Further research on how the heterozygous R446H variant contributes to VWM is required in the future.

## Data Availability

The original contributions presented in the study are included in the article/Supplementary Material; further inquiries can be directed to the corresponding author.
