# Supplementary material for: Identification of a Novel Heterozygous Mutation in the EIF2B4 Gene Associated With Vanishing White Matter Disease
Source: Front Bioeng Biotechnol. 2022 Jul 4;10:901452. doi: 10.3389/fbioe.2022.901452 (PMC9289103; doi:10.3389/fbioe.2022.901452)
Supplement: Supplementary file 1 [file DataSheet1.docx]

**Supplementary Material**

**Table S1. 160 genes targeted for capture and sequencing.**

| Gene | NM number | Chromosome | Exons | Gene | NM number | Chromosome | Exons |
| --- | --- | --- | --- | --- | --- | --- | --- |
| AARS2 | NM_020745 | Chr6 | 22 | *MMUT* | NM_000255 | Chr6 | 13 |
| ABAT | NM_001127448 | Chr16 | 16 | *NDUFA1* | NM_004541 | ChrX | 3 |
| ABCD1 | NM_000033 | ChrX | 10 | *NDUFA10* | NM_004544 | Chr2 | 10 |
| ACOX1 | NM_004035 | Chr17 | 14 | *NDUFA11* | NM_175614 | Chr19 | 4 |
| ACTA2 | NM_001613 | Chr10 | 9 | *NDUFA12* | NM_018838 | Chr12 | 4 |
| ADH1C | NM_000669 | Chr4 | 9 | *NDUFA2* | NM_002488 | Chr5 | 3 |
| AIMP1 | NM_004757 | Chr4 | 7 | *NDUFA9* | NM_005002 | Chr12 | 11 |
| ALDH3A2 | NM_000382 | Chr17 | 10 | *NDUFAF1* | NM_016013 | Chr15 | 5 |
| *APBB2* | NM_004307 | Chr4 | 18 | *NDUFAF2* | NM_174889 | Chr5 | 4 |
| *ARSA* | NM_001085425 | Chr22 | 8 | *NDUFAF3* | NM_199069 | Chr3 | 5 |
| *ASPA* | NM_000017 | Chr17 | 7 | *NDUFAF4* | NM_014165 | Chr6 | 3 |
| *BCKDHA* | NM_000709 | Chr19 | 9 | *NDUFAF6* | NM_152416 | Chr8 | 9 |
| *BCKDHB* | NM_000056 | Chr6 | 11 | *NDUFB3* | NM_002491 | Chr2 | 3 |
| *BCS1L* | NM_004328 | Chr2 | 9 | *NDUFS1* | NM_005006 | Chr2 | 19 |
| *CHM* | NM_000390 | ChrX | 15 | *NDUFS2* | NM_004550 | Chr1 | 15 |
| *CLCN2* | NM_004366 | Chr3 | 24 | *NDUFS3* | NM_004551 | Chr11 | 7 |
| *COA5* | NM_001008215 | Chr2 | 3 | *NDUFS4* | NM_002495 | Chr5 | 5 |
| *COX15* | NM_004376 | Chr10 | 9 | *NDUFS6* | NM_004553 | Chr5 | 4 |
| *COX6B1* | NM_001863 | Chr19 | 4 | *NDUFS7* | NM_024407 | Chr19 | 8 |
| *CSF1R* | NM_005211 | Chr5 | 22 | *NDUFS8* | NM_002496 | Chr11 | 7 |
| *CTC1* | NM_025099 | Chr17 | 23 | *NDUFV1* | NM_007103 | Chr11 | 10 |
| *CYP27A1* | NM_000784 | Chr2 | 9 | *NDUFV2* | NM_021074 | Chr18 | 8 |
| *DARS2* | NM_018122 | Chr1 | 17 | *NOS3* | NM_000603 | Chr7 | 20 |
| *DBT* | NM_001918 | Chr1 | 11 | *NOTCH3* | NM_000435 | Chr19 | 33 |
| *DDC* | NM_000790 | Chr7 | 15 | *NR0B1* | NM_000475 | ChrX | 2 |
| *DLD* | NM_000108 | Chr7 | 14 | *NUBPL* | NM_025152 | Chr14 | 11 |
| *DNAJC13* | NM_015268 | Chr3 | 20 | *OCRL* | NM_000276 | ChrX | 20 |
| *DNAJC6* | NM_014787 | Chr1 | 9 | *PARK2* | NM_004562 | Chr6 | 14 |
| *ECM1* | NM_004425 | Chr1 | 9 | *PARK7* | NM_001123377 | Chr1 | 7 |
| *EIF2B1* | NM_001414 | Chr12 | 9 | *PAXIP1* | NM_007349 | Chr7 | 21 |
| *EIF2B2* | NM_014239 | Chr14 | 8 | *PC* | NM_000920 | Chr11 | 22 |
| *EIF2B3* | NM_020365 | Chr1 | 12 | *PDGFRB* | NM_002609 | Chr5 | 23 |
| *EIF2B4* | NM_001034116 | Chr2 | 13 | *PEX1* | NM_000466 | Chr7 | 24 |
| *EIF2B5* | NM_003907 | Chr3 | 16 | *PEX10* | NM_002617 | Chr1 | 6 |
| *EIF4G1* | NM_004953 | Chr3 | 20 | *PEX12* | NM_000286 | Chr17 | 3 |
| *ERCC6* | NM_000124 | Chr10 | 21 | *PEX13* | NM_002618 | Chr2 | 4 |
| *ERCC8* | NM_00082 | Chr5 | 12 | *PEX14* | NM_004565 | Chr1 | 9 |
| *ETFA* | NM_000126 | Chr15 | 12 | *PEX16* | NM_004813 | Chr11 | 11 |
| *ETFB* | NM_001985 | Chr19 | 6 | *PEX19* | NM_002857 | Chr1 | 8 |
| *ETFDH* | NM_004453 | Chr4 | 13 | *PEX2* | NM_000318 | Chr8 | 4 |
| *ETHE1* | NM_001320867 | Chr19 | 7 | *PEX26* | NM_017929 | Chr22 | 6 |
| *FA2H* | NM_024306 | Chr16 | 7 | *PEX3* | NM_003630 | Chr6 | 12 |
| *FAM126A* | NM_001363466 | Chr7 | 12 | *PEX5* | NM_000319 | Chr12 | 16 |
| *FASTKD2* | NM_014929 | Chr2 | 12 | *PEX6* | NM_000287 | Chr6 | 17 |
| *FKTN* | NM_006731 | Chr9 | 10 | *PEX7* | NM_000288 | Chr6 | 10 |
| *FOLR1* | NM_000802 | Chr11 | 5 | *PINK1* | NM_032409 | Chr1 | 8 |
| *FOXRED1* | NM_017547 | Chr11 | 11 | *PLA2G6* | NM_003560 | Chr22 | 17 |
| *FUCA1* | NM_000147 | Chr1 | 8 | *PLAU* | NM_002658 | Chr10 | 11 |
| *GABRG2* | NM_000816 | Chr5 | 9 | *PLP1* | NM_000533 | ChrX | 7 |
| *GALC* | NM_000153 | Chr14 | 17 | *POLR3A* | NM_007055 | Chr10 | 12 |
| *GALE* | NM_000403 | Chr1 | 12 | *POLR3B* | NM_018082 | Chr12 | 20 |
| *GALK1* | NM_000154 | Chr17 | 8 | *PSAP* | NM_002778 | Chr10 | 14 |
| *GALT* | NM_001258332 | Chr9 | 9 | *PSEN2* | NM_000447 | Chr1 | 13 |
| *GAN* | NM_001377486 | Chr16 | 10 | *PTCH1* | NM_000264 | Chr9 | 24 |
| *GBA* | NM_000157 | Chr1 | 11 | *RASA1* | NM_002890 | Chr5 | 20 |
| *GFAP* | NM_001131019 | Chr17 | 8 | *RNASEH2A* | NM_006397 | Chr19 | 8 |
| *GIGYF2* | NM_015575 | Chr2 | 20 | *RNASEH2B* | NM_024570 | Chr13 | 11 |
| *GJB1* | NM_001097642 | ChrX | 2 | *PRNP* | NM_000311 | Chr20 | 2 |
| *GJC2* | NM_020435 | Chr1 | 2 | *RNASEH2C* | NM_032193 | Chr11 | 4 |
| *GLB1* | NM_001079811 | Chr3 | 16 | *RNASET2* | NM_003730 | Chr6 | 9 |
| *HEPACAM* | NM_152722 | Chr11 | 7 | *SAMHD1* | NM_015474 | Chr20 | 16 |
| *HFE* | NM_000410 | Chr6 | 6 | *SCP2* | NM_002979 | Chr1 | 16 |
| *HSPD1* | NM_002156 | Chr2 | 12 | *SDHA* | NM_004168 | Chr5 | 15 |
| *HTRA1* | NM_002775 | Chr10 | 9 | *SDHAF1* | NM_001042631 | Chr19 | 1 |
| *HTRA2* | NM_013247 | Chr2 | 8 | *SLC16A2* | NM_006517 | ChrX | 6 |
| *ISG15* | NM_005101 | Chr1 | 2 | *SLC19A3* | NM_025246 | Chr2 | 6 |
| *L2HGDH* | NM_024884 | Chr14 | 10 | *SLC20A2* | NM_006749 | Chr8 | 11 |
| *LMBRD1* | NM_018368 | Chr6 | 16 | *SNCA* | NM_000345 | Chr4 | 6 |
| *LMNB1* | NM_005573 | Chr5 | 11 | *SOX10* | NM_006941 | Chr22 | 4 |
| *MBP* | NM_001025081 | Chr18 | 7 | *SUMF1* | NM_182760 | Chr3 | 9 |
| *MCCC1* | NM_020166 | Chr3 | 19 | *SURF1* | NM_003172 | Chr9 | 9 |
| *MGP* | NM_001190839 | Chr12 | 5 | *TBP* | NM_003194 | Chr6 | 8 |
| *MLC1* | NM_015166 | Chr22 | 12 | *TREM2* | NM_018965 | Chr6 | 5 |
| *MMAA* | NM_172250 | Chr4 | 7 | *TREX1* | NM_007248 | Chr3 | 2 |
| *MMAB* | NM_052845 | Chr12 | 9 | *TUBB4A* | NM_001289123 | Chr19 | 5 |
| *MMACHC* | NM_015506 | Chr1 | 4 | *TUFM* | NM_001365360 | Chr16 | 10 |
| *MMADHC* | NM_015702 | Chr2 | 8 | *TYMP* | NM_001953 | Chr22 | 10 |
| *MPV17* | NM_002437 | Chr2 | 8 | *TYROBP* | NM_003332 | Chr19 | 5 |
| *MPZ* | NM_000530 | Chr1 | 6 | *UCHL1* | NM_004181 | Chr4 | 9 |
| *MTRR* | NM_001364440 | Chr5 | 15 | *XPR1* | NM_004736 | Chr1 | 15 |

**Figure S1. Sanger sequence results of unaffected-family members.**

**

**
